# Metabolomics Comparison of Drug-Resistant and Drug-Susceptible *Pseudomonas aeruginosa* Strain (Intra- and Extracellular Analysis)

**DOI:** 10.3390/ijms221910820

**Published:** 2021-10-06

**Authors:** Karolina Anna Mielko, Sławomir Jan Jabłoński, Łukasz Pruss, Justyna Milczewska, Dorota Sands, Marcin Łukaszewicz, Piotr Młynarz

**Affiliations:** 1 Department of Biochemistry, Molecular Biology and Biotechnology, Faculty of Chemistry, Wroclaw University of Science and Technology, 50-373 Wroclaw, Poland; karolina.mielko@pwr.edu.pl (K.A.M.); lukasz.pruss@pwr.edu.pl (Ł.P.); 2 Biotransformation Department, Faculty of Biotechnology, University of Wroclaw, 50-383 Wroclaw, Poland; slawomir.jablonski@uwr.edu.pl (S.J.J.); marcin.lukaszewicz@uwr.edu.pl (M.Ł.); 3 Ardigen, 30-394 Kraków, Poland; 4 Cystic Fibrosis Department, Institute of Mother and Child, 01-211 Warsaw, Poland; justyna.milczewska@imid.med.pl (J.M.); dorota.sands@imid.med.pl (D.S.)

**Keywords:** *Pseudomonas aeruginosa*, metabolomics, antibiotic resistance, NMR spectroscopy

## Abstract

*Pseudomonas aeruginosa* is a common human pathogen belonging to the ESKAPE group. The multidrug resistance of bacteria is a considerable problem in treating patients and may lead to increased morbidity and mortality rate. The natural resistance in these organisms is caused by the production of specific enzymes and biofilm formation, while acquired resistance is multifactorial. Precise recognition of potential antibiotic resistance on different molecular levels is essential. Metabolomics tools may aid in the observation of the flux of low molecular weight compounds in biochemical pathways yielding additional information about drug-resistant bacteria. In this study, the metabolisms of two *P. aeruginosa* strains were compared—antibiotic susceptible vs. resistant. Analysis was performed on both intra- and extracellular metabolites. The ^1^H NMR method was used together with multivariate and univariate data analysis, additionally analysis of the metabolic pathways with the FELLA package was performed. The results revealed the differences in *P. aeruginosa* metabolism of drug-resistant and drug-susceptible strains and provided direct molecular information about *P. aeruginosa* response for different types of antibiotics. The most significant differences were found in the turnover of amino acids. This study can be a valuable source of information to complement research on drug resistance in *P. aeruginosa*.

## 1. Introduction

*Pseudomonas aeruginosa* is a Gram-negative opportunistic human pathogen [1], which causes infections in chronic wounds and in the urinary tract. Moreover, it is responsible for respiratory tract infections in cystic fibrosis (CF), obstructive lung disease, or mechanically ventilated patients [2]. As a part of the ESKAPE pathogens group (*Enterococcus faecium*, *Staphylococcus aureus*, *Klebsiella pneumoniae*, *Acinetobacter baumannii*, *Pseudomonas aeruginosa*, and *Enterobacter species*), *P. aeruginosa* is a significant cause of nosocomial infections. In some *P. aeruginosa* strains, antibiotic therapy is not successful, despite its sensitivity in laboratory tests [3,4]. Due to the increasing number of multidrug-resistant (MDR) isolates, the WHO has recognized *P. aeruginosa* as a priority pathogen for antibiotic research [5]. The development of new antibacterial therapies and understanding antibiotic resistance mechanisms is crucial for clinical practice and finding new treatment possibilities [6,7,8].

Multidrug resistance of *P. aeruginosa* relies on several mechanisms: (i) antibiotic molecules may be neutralized by specialized enzymes (β-lactams, aminoglycosides), (ii) therapeutic compounds may also be removed from the cell by efflux pumps (β-lactams, quinolones, and some aminoglycosides), (iii) alteration of the antibiotic target molecule, (iv) modifications in the penicillin-binding proteins (PBPs), and (v) changes in OPrD porin [4,9,10,11,12].

An important factor affecting drug efficiency is drug accessibility for targeted microorganisms. Biofilm produced by *P. aeruginosa* is a physical barrier protecting bacteria from antibiotics [13]. Some *P. aeruginosa* strains exhibit decreased expression of genes encoding the porins (outer membrane channel proteins) used by certain types of antibiotics (e.g., rifamycins and quinolones) to enter the cell [11]. Antibiotic intake may also be reduced due to reduced membrane potential [14]. These *P. aeruginosa* attributes are important factors that should be considered during the development of an efficient antibiotic therapy.

Bacterial cells can become antibiotic-resistant when stringent response (SR) is activated due to the nutrient limitation resulting in reduced bacterial multiplication. The correct activity of SR is also crucial for biofilm development and stability [15].

The drug resistance phenomenon is analyzed on many levels: genomic, transcriptomic, proteomic, and metabolomics. The last link of this omics chain may provide information on antibiotic resistance mechanisms [16]. Metabolomics is focused on the analysis and monitoring of low molecular weight compounds involved in cell metabolism. Metabolomic studies performed on microorganisms so far usually rely on chromatographic techniques coupled with mass spectrometry (MS) and nuclear magnetic resonance spectroscopy (NMR) [17,18].

Metabolomics experiments have enabled the identification of new metabolic pathways [18], the recognition of bacterial strain origin [19], the identification of microorganism species [20,21], and the analysis of the influence of different external factors on bacteria [22]. Moreover, metabolomic and genomic analysis helped explain *P. aeruginosa* polymyxin resistance [23]. Metabolome analysis techniques are also considered an advanced diagnostic tool for bacterial infections [24].

The presence of some antibiotic resistance mechanisms may be correlated with changes in metabolite concentration (inside and outside the cell). Resistance to some antibiotics is associated with the presence and activity of proteins involved in the processing of certain metabolites in the cell (oprD protein required for carbapenem uptake is an amino acid transporter [10] and activator of SR, which results in changes in the expression of the enzymes involved in the main metabolic pathways including amino acid synthesis [25]). Metabolome analysis would give additional information alongside antibiotic sensitivity tests. This may help in the choice of appropriate antibiotic therapy, as well as enabling research in new therapeutic strategies [26].

The metabolomics comparison of strains with different antibiotic resistance could give information about the differences in the phenotype of both strains and may show the direction of further (more detailed) investigations. The analysis of intra- and extracellular metabolites allows us to observe the differences in intracellular machinery of the bacteria, but also the association with the bacterial environment. All these reasons can give additional information for transcriptomics and genomics studies and may enable the recognition of more antibiotic resistance mechanisms in *P. aeruginosa.* This study aimed to delineate metabolic differences between two *P. aeruginosa* strains isolated from CF patients: a strain resistant to the majority of available antibiotics (except colistin and ciprofloxacin) and an antibiotic susceptible strain, by use of metabolomics tools using the ^1^H NMR method together with univariate and multivariate analysis of intra- and extracellular metabolites and the application of bioinformatics metabolic pathway analysis software (FELLA).

## 2. Results

### 2.1. Antibiotic Resistance Test

The results of antibiotic resistance tests are presented in Table 1. In this experiment, antibiotics from the following groups: aminoglycoside (disrupting protein synthesis by binding to the 30S ribosomal subunit), beta-lactams (disrupting peptidoglycan biosynthesis), quinolone (disrupting DNA replication), and polymyxin (disrupting cell membrane) were used. Strain PAW17 was susceptible to all tested antibiotics. Strain PAW23 was susceptible only to two antibiotics: ciprofloxacin and colistin.

### 2.2. Metabolites Identification

#### 2.2.1. Intracellular Metabolites

In total, 32 intracellular metabolites were identified (5-aminopentanoate, acetate, adenine, alanine, AMP, aspartate, betaine, ethanol, formate, glucose, glutamate, glycine, histamine, histidine, homoserine, isobutyrate, isocitrate, isoleucine, lactate, leucine, methionine, NAD+, oxypurinol, phenylalanine, pyruvate, sarcosine, succinate, threonine, tyrosine, UMP, uracil, and valine). Information about the chemical shift for each metabolite is available in Supplementary Materials (Table S1). The set of identified metabolites was identical for both strains.

**Figure 1:**
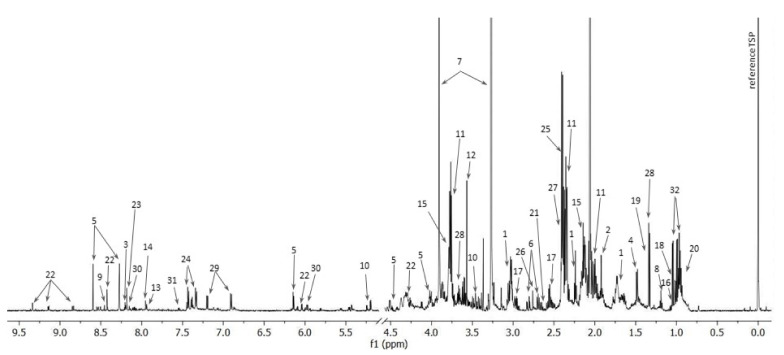
The representative 1D ^1^H NMR spectrum of intracellular metabolites of drug-susceptible *Pseudomonas aeruginosa* strain. (1—5-aminopentanoate, 2—acetate, 3—adenine, 4—alanine, 5—AMP, 6—aspartate, 7—betaine, 8—ethanol, 9—formate, 10—glucose, 11 -glutamate, 12—glycine, 13—histamine, 14—histidine, 15—homoserine, 16—isobutyrate, 17—isocitrate, 18—isoleucine, 19—lactate, 20—leucine, 21—methionine, 22—NAD+, 23—oxypurinol, 24—phenylalanine, 25—pyruvate, 26—sarcosine, 27—succinate, 28—threonine, 29—tyrosine, 30—UMP, 31—uracil, 32—valine).

The representative ^1^H NMR spectrum of intracellular metabolites is presented below (Figure 1).

#### 2.2.2. Intracellular Metabolites

In total, 27 extracellular metabolites were identified (6-hydroxynicotinate, acetate, alanine, betaine, formate, glycine, histamine, histidine, imidazole, isobutyrate, glutamate, aspartate, asparagine, pyroglutamate, isoleucine, leucine, lysine, methanol, methionine, oxypurinol, phenylalanine, pyruvate, threonine, trehalose, tryptophan, tyrosine, and valine). Four metabolites were not present in the post-culture medium (glutamate, aspartate, asparagine, and pyroglutamate). Information about the chemical shift for each metabolite is available in Supplementary Materials (Table S2).

**Figure 2:**
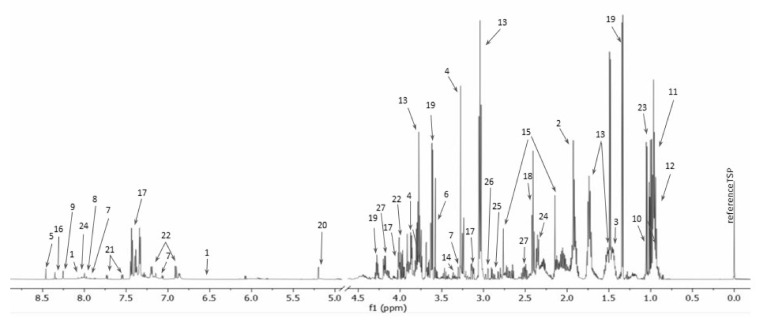
The representative 1D ^1^H NMR spectrum of extracellular metabolites of drug-susceptible *Pseudomonas aeruginosa* strain (1—6-hydroxynicotinate; 2—acetate; 3—alanine; 4—betaine; 5—formate; 6—glycine; 7—histamine; 8—histidine; 9—imidazole; 10—isobutyrate; 11—isoleucine; 12—leucine; 13—lysine; 14—methanol; 15—methionine; 16—oxypurinol; 17—phenylalanine; 18—pyruvate; 19—threonine; 20—trehalose; 21—tryptophan; 22—tyrosine; 23—valine; 24—glutamate; 25—aspartate; 26—asparagine; 27—pyroglutamate).

The representative ^1^H NMR spectrum of intracellular metabolites is presented in (Figure 2).

The total number of intra- and extracellular metabolites identified was 39. Among these, 19 metabolites were common to intra- and extracellular environments (histidine, aspartate, glutamate, histamine, alanine, pyruvate, valine, isoleucine, leucine, betaine, methionine, formate, glycine, threonine, phenylalanine, tyrosine, isobutyrate, oxypurinol, and acetate); 13 metabolites were identified in cell extracts (succinate, homoserine, lactate, UMP, sarcosine, ethanol, isocitrate, adenine, glucose, NAD+, AMP, 5-aminopentanoate, and uracil) and eight were identified only in the culture medium (imidazole, asparagine, methanol, 6-hydroxynicotinate, pyroglutamate, tryptophan, lysine, and trehalose). 

### 2.3. Multivariate Data Analysis

**Figure 3:**
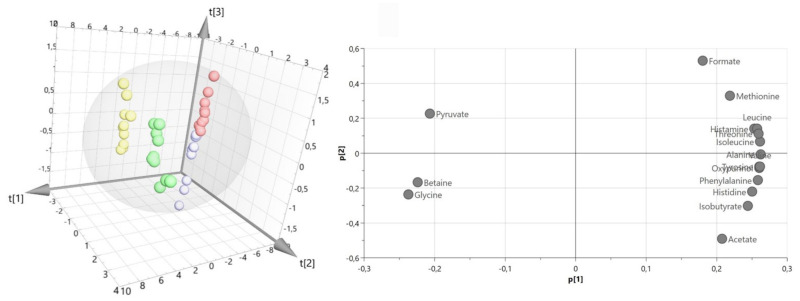
PCA model plot and corresponding loading plot for of ^1^H NMR data of all metabolites of *P. aeruginosa* strains (drug-resistant extracellular (green), drug-susceptible extracellular (yellow), drug-resistant intracellular (blue), and drug-susceptible intracellular (red)). Symbols in the same color represent biological repetitions.

The performed PCA—multivariate unsupervised analysis between drug-resistant and drug-susceptible isolates revealed the natural grouping between bacterial intra- and extracellular metabolites (Figure 3). Direct comparison of intracellular metabolites revealed a more similar metabolomics profile than between extracellular metabolites. The first three principal components (PC) accounted respectively for 84.7%, 8.74%, and 2.56% of the total variance in the data (R2X = 0.998). The obtained loading plots analysis showed three metabolites differentiated between bacteria cells and medium, which are: glycine, betaine, and pyruvate. 

Supervised analysis OPLS-DA analysis provides the strain’s grouping. The CV-ANOVA test of this model gave statistically important results (model with parameters are available in Supplementary Materials (Figure S1)).

#### 2.3.1. Intracellular Metabolites

**Figure 4:**
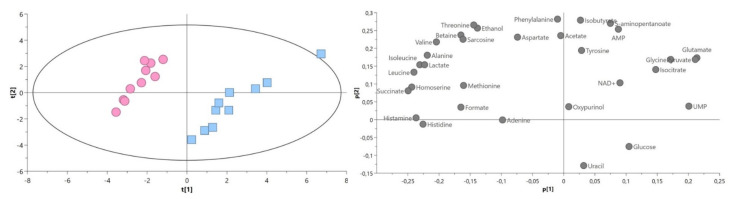
PCA model plot and corresponding loading plot of ^1^H NMR data of intracellular metabolites of drug-resistant (blue squares) and drug-susceptible (red circle) *P. aeruginosa* strains. Symbols in the same color represent biological repetitions.

PCA score plot revealed the clustering of bacterial isolates with and without antibiotic resistance (Figure 4). The first and second principal components (PC) accounted, respectively, for 41.6% and 21.5% of the total variance in the data (R2X = 0.631).

Supervised OPLS-DA analysis provides the strain’s grouping. The CV-ANOVA test of this model gave statistically important results (model with parameters are available in Supplementary Materials (Figure S2)).

#### 2.3.2. Extracellular Metabolites

For the PCA extracellular metabolites analysis, in addition to two types of bacterial strain isolates, a control group was also taken into consideration (the entire content of medium before bacterial cultivation).

**Figure 5:**
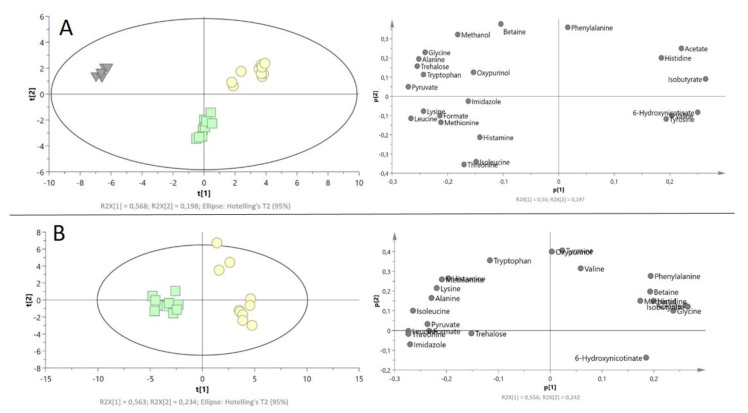
PCA model plots and corresponding loading plots for ^1^H NMR data of extracellular metabolites of *P. aeruginosa* strains. (**A**) Both *P. aeruginosa* strains with control; (**B**) both *P. aeruginosa* strains. Drug-resistant extracellular (green squares), drug-susceptible extracellular (yellow circle), control—LB medium (gray triangles). Symbols in the same color represent biological repetitions.

The PCA score plot showed the difference between sample distributions for bacteria isolates with different antibiotic resistance, while the control group formed a separate data set. In a comparison of both *P. aeruginosa* strains with control, the first and second principal components (PC) accounted for 56.0% and 19.7% of the total variance in the data, respectively, (R2X = 0.996) (Figure 5A). The score plot showed the differences between the amino acids (glycine, alanine, tryptophan, leucine, lysine, methionine, threonine, and histidine), pyruvate, isobutyrate, acetate, tyrosine, and 6-hydroxynicotinate.

The direct comparison between the *P. aeruginosa* strains showed a clear separation between the studied groups. The first and second principal components (PC) accounted, respectively, for 55.6% and 23.2% of the total variance. Differences were mainly observed in amino acid levels (Figure 5B)**.**

For each comparison, the supervised OPLS-DA analysis provides the strain’s grouping. The CV-ANOVA test of this model gave statistically important results (model with parameters are available in Supplementary Materials (Figure S3)). Information concerning an additional PCA single comparison of the drug-resistant *P. aeruginosa* strain with control and drug-susceptible *P. aeruginosa* strain with control is available in Supplementary Materials (Figure S4).

### 2.4. Statistical Analysis

#### 2.4.1. Intracellular Metabolites

Among all the identified metabolites, 20 showed a statistically significant difference between susceptible and resistant strains (*p* < 0.05) (succinate, homoserine, histidine, histamine, lactate, alanine, glutamate, pyruvate, UMP, valine, isoleucine, leucine, betaine, methionine, formate, glycine, sarcosine, threonine, ethanol, and isocitrate).

In the same comparison, VIP scores greater than 1.00 were obtained for 16 overlapped metabolites (all statistically important metabolites, without glycine, threonine, succinate, and lactate). Detailed statistical data are shown in Table 2.

**Figure 6:**
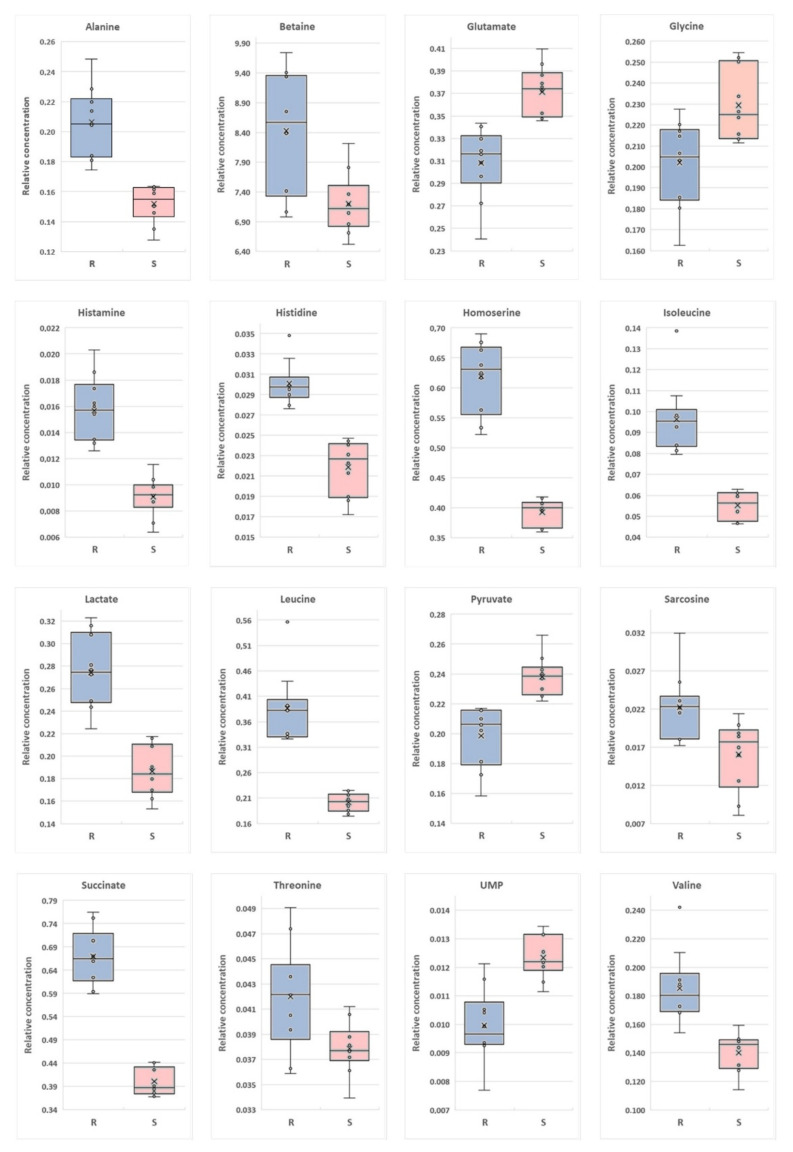
Boxplots for intracellular metabolites with VIP (Variable Importance in Projection) scores above 1.00 are statistically important after *p*-value adjustment (*q* < 0.05). Red bars—S—drug-susceptible strain; blue bars—R—drug-resistant strain.

Among the differentiating metabolites the relative concentration of succinate, homoserine, lactate, alanine, valine, isoleucine, leucine, betaine, histidine, histamine, methionine, formate, sarcosine, threonine, and ethanol were upregulated in the group of drug-resistant *P. aeruginosa*, while only five metabolites were at the higher-level for drug-sensitive samples—glutamate, pyruvate, UMP, glycine, isocitrate (Figure 6).

#### 2.4.2. Extracellular Metabolites

The comparison of the drug-resistant *P. aeruginosa* strain with the drug-susceptible strain enabled the identification of 16 statistically significant metabolites (*p* < 0.05) (acetate, alanine, betaine, glycine, formate, histidine, imidazole, isobutyrate, isoleucine, leucine, lysine, methanol, phenylalanine, pyruvate, threonine and 6-hydroxynicotinate), while a VIP scores greater than 1.00 were found for nine metabolites (acetate, glycine, imidazole, isobutyrate, isoleucine, leucine, phenylalanine, pyruvate, and threonine).

The comparison of the drug-resistant *P. aeruginosa* strain with the control (LB medium) enabled the identification of 17 statistically significant metabolites (*p* < 0.05) (acetate, alanine, betaine, glycine, isobutyrate, leucine, lysine, methanol, methionine, phenylalanine, pyruvate, trehalose, threonine, tryptophan, tyrosine, valine and 6-hydroxynicotinate). In this case, VIP scores greater than 1.00 were obtained for 15 metabolites (all statistically important metabolites, without acetate, and threonine).

The comparison of the drug-susceptible *P. aeruginosa* strain with the control (LB medium) identified 19 statistically significant metabolites (*p* < 0.05) (acetate, alanine, betaine, glycine, histidine, isobutyrate, isoleucine, leucine, lysine, methanol, methionine, phenylalanine, pyruvate, trehalose, threonine, tryptophan, tyrosine, valine and 6-hydroxynicotinate). 14 metabolites had VIP score greater than 1.00 (all statistically significant metabolites, without isoleucine, histidine, methionine, imidazole, and betaine). Detailed statistical data are available in Table 3.

**Figure 7:**
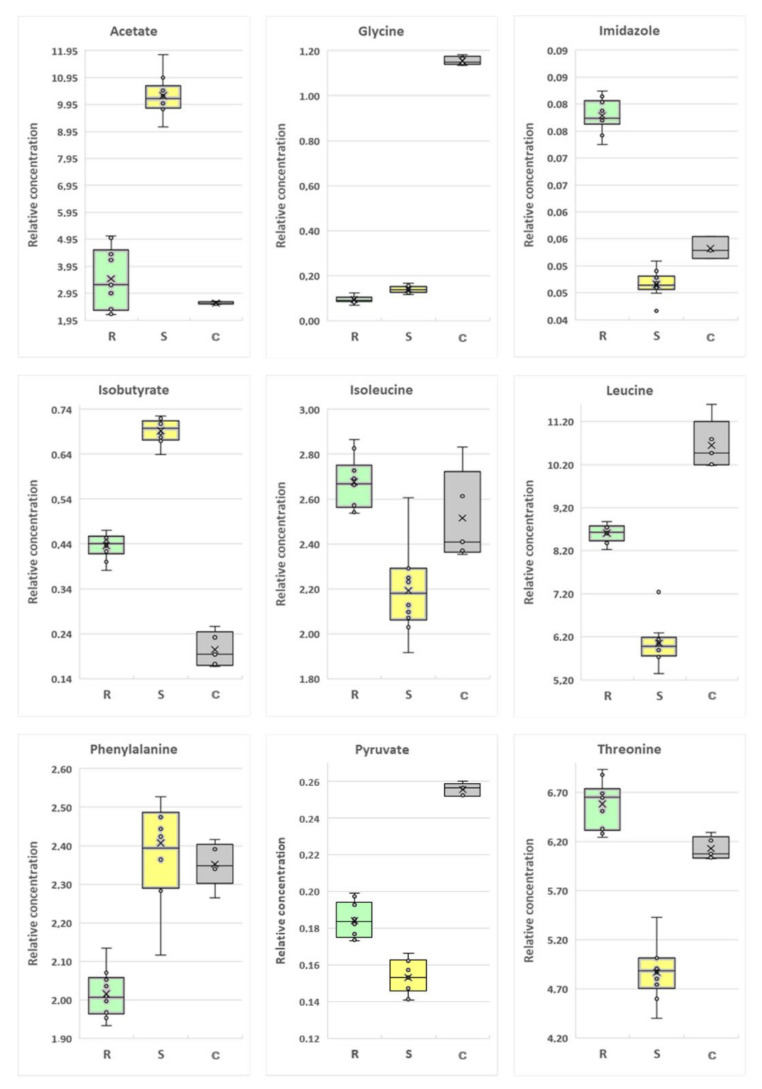
Boxplots for extracellular metabolites with VIP (Variable Importance in Projection) scores above 1.00 and statistically important after *p*-value adjustment (*q* < 0.05). Yellow bars—S—drug-susceptible strain. green bars—R—drug-resistant strain, gray bars—C—control (medium LB).

Among the differentiating metabolites, the relative concentration of alanine, formate, imidazole, isoleucine, leucine, lysine, pyruvate, and threonine was upregulated in the group of drug-resistant *P. aeruginosa* vs. drug-susceptible strains. Eight metabolites were at a higher level in drug-susceptible samples—6-hydroksynicotinate, acetate, betaine, glycine, histidine, isobutyrate, methanol, and phenylalanine. The relative concentration of three metabolites was lower in bacterial culture media than the control medium—glycine, leucine, and pyruvate. The concentrations of isobutyrate and acetate were higher in cell culture media than in control samples (Figure 7).

### 2.5. Bioinformatics Analysis

Bioinformatics analysis was used to produce a graphical representation of compounds, enzymes, reactions, modules, and pathways with information on how the input metabolites found in the statistical analysis reach the suggested pathways and on how these pathways cross-talk. An interactive version of the graphic for analysis is also available. For this analysis metabolites with statistical importance and VIPs greater than 1.00 were chosen.

#### 2.5.1. Intracellular Metabolites

**Figure 8:**
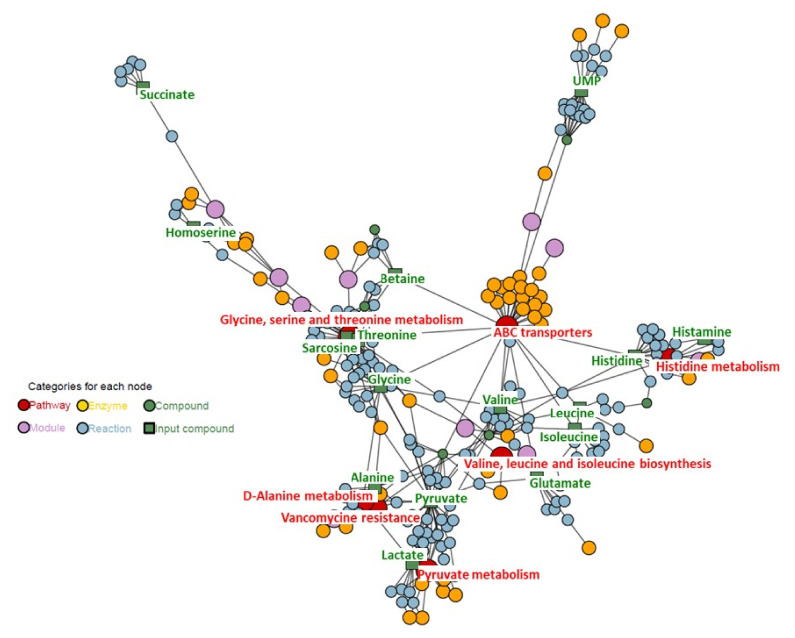
Results of the node prioritization by FELLA in the *Pseudomonas aeruginosa* strain (intracellular analysis).

The graphical representation of compounds, enzymes, reactions, modules, pathways, with information on how the input metabolites reach the suggested pathways, and on how these pathways cross-talk for intracellular metabolites is shown in Figure 8.

The main pathways connected with these compounds are ABC transporters, vancomycin metabolism pathway, and amino acids pathways, especially glycine, serine, threonine, alanine, and pyruvate metabolism.

#### 2.5.2. Extracellular Metabolites

**Figure 9:**
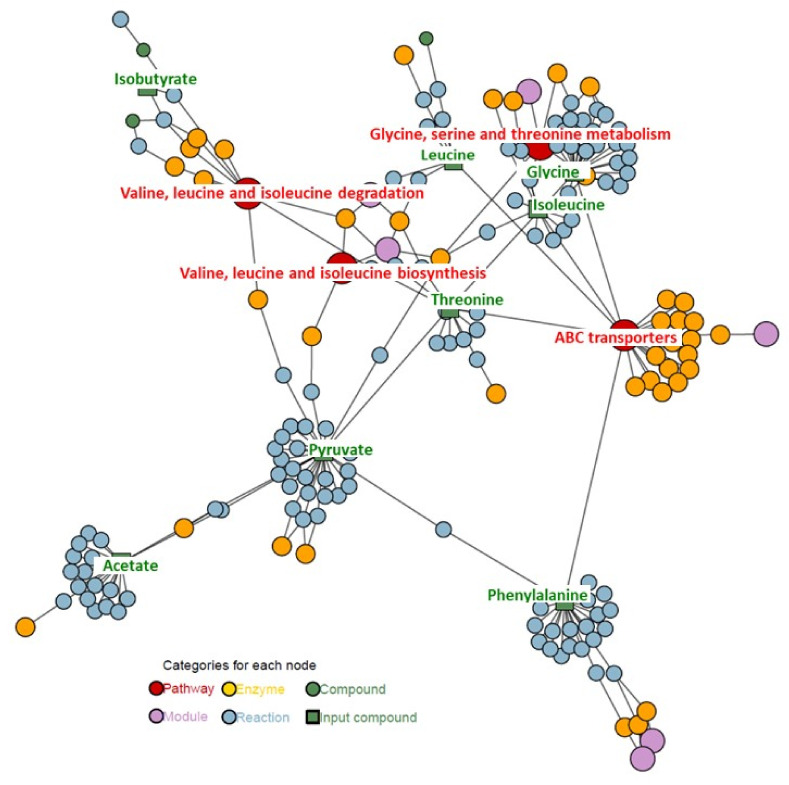
Results of the node prioritization by FELLA in the *Pseudomonas aeruginosa* strain (extracellular analysis).

The graphical representation of compounds, enzymes, reactions, modules, pathways, and information on how the input metabolites reach the suggested pathways, and on how these pathways cross-talk for extracellular metabolites is shown in Figure 9.

The main pathways connected with these compounds are ABC transporters, vancomycin metabolism pathway, and amino acids pathways.

The interactive versions of Figure 8 and Figure 9 are available in Supplementary Materials (Files S1 and S2).

## 3. Discussion

Increasing antibiotic resistance leads to the development of alternative chemotherapeutics dedicated to treating bacterial infections. In the case of *P. aeruginosa* infections, treatment with β-lactams, fluoroquinolones, and aminoglycosides is widely used. In multi-drug resistant strains, ceftolozan/tazobactam and colistin could also be administered, but these can cause toxic and adverse side effects [3,4,12]. New therapeutic strategies may involve the addition of compounds improving the activity of traditional antibiotics [27]. Understanding the exact mechanisms of action of currently used drugs could support this process, which is why it is so important to perform analyses at various molecular levels [8]. Insights about differences between drug-resistant and drug-sensitive strains can be given by metabolomics studies, where the differences in the low-molecular-weight compounds’ regulation reflect the changes in biochemical pathways [28]. This approach may be helpful for the delineation of molecular targets and drug design [29]. In addition, bacteriostatic compounds based on new structures and mechanisms of action are being designed [30]. Information regarding the prevalence of antibiotic resistance mechanisms would support the development process.

However, these strategies will probably not be universal. In such a situation, selection of effective therapy should be based on a detailed analysis of resistance mechanisms present in an individual clinical strain. Treatment of antibiotic-resistant bacteria is likely to require an individual approach. Therefore, the development of a fast and accurate diagnostic tool seems to be necessary. Our results show that the metabolomics approach with the use of ^1^H NMR spectroscopy may establish new horizons in this area [29].

The analysis of all accessible sources of data may provide an interesting insights into the evolutionary adaptation of *P. aeruginosa*. Among intracellular metabolites there is an interesting relationship between pyruvate and its reduction product—lactate. In the antibiotic resistant strain, the level of pyruvate was decreased while the level of lactate is increased, in comparison to antibiotic-susceptible strain. This may be the result of a more intense reduction reaction of pyruvate in the drug-resistant strain since none of the strains secreted these compounds to the culture medium. However, research in cystic fibrosis (CF) patients’ samples shows that lactate is a major component in sputum, and could be an important infection factor [31]. Pyruvate seems also to be associated with BCAA biosynthesis (valine, leucine, and isoleucine), which were upregulated in the drug-resistant strain [32]. Moreover, significant differences were observed in the intracellular concentrations of isocitrate (lower in the resistant strain) and succinate (higher in the resistant strain).

In the majority of cases, the identified intracellular amino acids were at the higher level in the case of the antibiotic-resistant strain. The exceptions were glycine, glutamate, and tyrosine whose concentrations were significantly higher in the antibiotic-resistant strain. According to literature data, the amino acid conversion pathways are more efficient in antibiotic-resistant strains [33]. Our results have shown clearly changes in amino acids in both strains. Furthermore, the metabolic modification to amino acids as carbon sources is essential in antibiotic resistance [14,34,35]. 

In the antibiotic resistance strain, the relative concentration of UMP was lower. The synthesis of UMP has been shown to play an important role in sustaining virulence, biofilm formation, and antibiotic resistance in *P. aeruginosa* [14,27]. The production of biofilm in bacteria creates a physical barrier and reduces the effectiveness of treatment with various antibiotics. Furthermore, biofilm-formation is a critical mechanism of adaptive resistance [2]. Our results seem to confirm this observation.

Analysis of culture media revealed that the tested strains differed in amino acid turnover patterns. Both strains used all available glutamate, aspartate, asparagine, and pyroglutamate. However, for the susceptible strain, some of metabolites were taken from the medium at a higher level: alanine, leucine, lysine, and methionine, while threonine, histamine, and isoleucine were mostly utilized only by the antibiotic susceptible strain. The common metabolite for both types of isolates is glycine which was drained from the cultivation medium and seems to be one of the crucial nutrients. Different amino acid utilization patterns may result from reduced expression of transport proteins. The FELLA analysis results suggest that amino acid uptake may be interrupted. The KEGG database includes only information regarding amino acid ABC-transporters localized in the inner membrane [36]. These proteins are not considered antibiotic transporters; however, their activity depends on the presence of porins transporting amino acids from the environment into the periplasmatic space (these relations are not included in KEGG). Porins (such as OprD) are recognized as structures necessary for antibiotic uptake. Our results suggest that resistance of a strain to some β-lactams may depend on the reduced expression of porin, also resulting in reduced amino acid uptake. Elevated glycine uptake in the case of the antibiotic-resistant strain may result from a lack of threonine absorption. To satisfy the cell threonine demand, the antibiotic-resistant strain probably synthesizes this amino acid from glycine [35,37]. Moreover, in *P. aeruginosa* enzymes required for sarcosine synthesis from glycine were identified [38]. The different amino acid metabolism could originate for three reasons: different protein turnover, influx–outflux equilibrium, or bacteria amino acid biosynthesis. This last phenomenon can be caused by all the bacterial organism biochemical machinery for proteinogenic amino acid synthesis [33,37].

In the culture medium, acetate and isobutyrate were also present. The concentration of both compounds was higher in the case of the antibiotic-susceptible strain. These compounds are products of the catabolic metabolism of amino acids. Increased concentration of acetate and isobutyrate may indicate more intense metabolic activity of the antibiotic susceptible strain. The concentration of acetate in the cultivation medium showed a negative correlation with infection length in CF patients [34]. Reduced production of acetate is considered a sign of adaptation of a pathogen to the environment in the respiratory tracts of CF patients.

Observed metabolic differences between examined strains may result from starvation response (SR) activation in the drug-resistant strain. SR relies on the presence of the ppGpp molecule produced by two enzymes relA and spoT. RelA becomes active when the cell suffers amino acid limitation. SpoT produces ppGpp in response to sugar, iron, and fatty acid deficiency. Both proteins were identified in *P. aeruginosa* [15]. SR activation influences gene expression involved in glycolysis, the TCA cycle, and the amino acid synthesis pathway. In *E. coli,* expression of the majority of enzymes for amino acid synthesis is upregulated [25]. Moreover, the expression of glyoxalate-producing enzymes is also increased. On the other hand, expression of enzymes producing oxalacetate from succinate is reduced. If SR mechanisms in *P. aeruginosa* cause a transcriptional response similar to the one observed in *E. coli,* altered enzyme expression may explain the observed changes in intracellular metabolite concentration.

## 4. Conclusions

The treatment of bacterial infections is a significant problem, especially when in pathogens form biofilm structures. Despite novel pharmacotherapy restoring CFTR functionality being available, CF patients still suffer from bacterial infections [39]. Therefore, research focused on the treatment of bacterial lung infections is still needed.

Our results show that the metabolic differences between antibiotic-resistant and antibiotic-susceptible bacteria strains may be linked with the activity of antibiotic resistance mechanisms. Comparison of intracellular and extracellular metabolite profiles showed differences between drug-resistant and drug-susceptible *P. aeruginosa* strains within the intracellular amino acid pool. The intracellular free amino acid concentration results from the balance between different processes: protein synthesis, uptake from the environment, and their biosynthesis and degradation. This information may be helpful in the selection of the most effective therapy and targets for future drugs.

## 5. Materials and Methods

### 5.1. Bacterial Strains and Culture Conditions

In this study, two *P. aeruginosa* strains were analyzed: PAW17 (antibiotic susceptible) and PAW23 (antibiotic resistant). The strains were isolated from patients suffering from CF in the Mother and Child Institute in Warsaw. For long-term storage, strains were kept as glycerol preserved suspensions at −80 °C.

After thawing from −80 °C, the bacteria were grown on Miller’s LB Broth agar (BioShop) overnight at 37 °C. In the next step, pre-culture was prepared. 5 mL of liquid LB medium in a test tube was inoculated with a single colony from the agar plate and incubated for 24 h at 37 °C with shaking (180 r.p.m.). After that, 100 mL of the culture in a 300 mL conical flask was prepared (initial OD_600nm_ = 0.1) and incubated for 24 h under the same conditions. The strains’ breeding for metabolomics analysis was performed without antibiotic treatment. After 24 h of cultivation, both strains were in the stationary phase.

To collect bacterial cells the culture was centrifuged (19,000 r.c.f., 5 min, 4 °C) (sigma 3–18 KS, Polygen), and the bacterial pellet was washed with 0.9% NaCl solution. Culture medium samples were stored at −80 °C. The bacterial pellets were lyophilized (ScanvacCoolsave, Labogene) and stored at −80 °C. Before extraction, each sample was weighted in tubes (Eppendorf). The entire protocol was repeated for each strain in ten biological repetitions. Additionally, to compare the levels of extracellular metabolites with fresh medium, five technical repetitions of fresh LB medium samples were analyzed.

### 5.2. Antibiotic Resistance

The susceptibility of *P. aeruginosa* strains to most antibiotics was determined by the disc diffusion method. The bacterial suspension with a density equal to 0.5 McFarland was inoculated with a swab on Mueller–Hinton II Agar. The following antibiotic discs were placed on the seeded medium: amikacin (30 µg), netilmicin (10 µg), tobramycin (10 µg), gentamicin (10 µg), ceftazidime (10 µg), cefepime (30 µg), imipenem (10 µg), meropenem (10 µg), levofloxacin (5 µg), piperacillin (30 µg), piperacillin / tazobactam (30/6 µg), ticarcillin / clavulanic acid (75/10 µg) (all Emapol antibiotic discs). The cultivation was carried out for 18 ± 2 h at 35 °C ± 1 °C under aerobic conditions. The results were interpreted following the current recommendations of the European Committee on Antimicrobial Susceptibility Testing (EUCAST) [40].

### 5.3. Extraction and Samples Preparation

#### 5.3.1. Intracellular Metabolites

20 mg of lyophilized cells were suspended in 600 µL of methanol and samples were disrupted for 5 min in TissueLyser (Tissue Lyser II, Qiagen, Venlo, Netherlands). Then 600 µL of water was added to each sample and again vortexed for 10 min. After the disintegration, samples were centrifuged for 10 min, at 12000 rpm at 4 °C (Micro 220R, Hettich), and 0.9 mL of clarified upper phase was transferred into a new tube. The extracts were evaporated in a vacuum centrifuge (WP-03, JW Electronic, United States) (40 °C, 1500 rpm, 10 h). In the next step, 600 µL of PBS buffer (0.1 M,10% D_2_O, pH = 7.0, TSP = 0.3 mM) was added to each sample and mixed for 1 min and 550 µL was transferred into NMR-tubes (SP, 5mm, Armar Chemicals, Germany) for measurements. Until the measurements were taken, the samples were stored at 4 °C.

#### 5.3.2. Extracellular Metabolites

After breeding, 1.5 mL medium were evaporated in a vacuum centrifuge (40 °C, 1500 rpm, 12 h). In the next step, 600 µL of PBS buffer (0.1 M (NaH_2_PO_4_/Na_2_HPO_4_),10% D_2_O, pH = 7.0, TSP = 0.5 mM) was added to each sample and mixed for 3 min and 550 µL was transferred into 5-mm NMR-tubes (5SP, Armar Chemicals, Germany) for measurements. Until the measurements were taken, the samples were stored at 4 °C.

### 5.4. ^1^H NMR Spectroscopy Analysis of the Bacterial Metabolites

Standard ^1^H NMR experiments were performed on a Bruker AVANCE II 600.58 MHz spectrometer (Bruker, GmBH, Germany) equipped with a 5 mm TBO probe at 298 K. All one-dimensional ^1^H NMR spectra were carried out using the cpmgpr1d (in Bruker notation) pulse sequence by suppression of water resonance by presaturation. Acquisition parameters were as follows: spectral width, 10 ppm; the number of scans, 128; acquisition time, 2.72 s per scan; relaxation delay, 3.5 s; and time-domain points, 64 K. The spectra were referenced to the TSP resonance at 0.0 ppm and manually corrected for phase and baseline (MestReNova v. 11.0.3, Qingdao, China).

### 5.5. Data Processing and Multivariate Statistical Data Analysis

All spectra were exported to Matlab (Matlab R2014a, v. 8.3.0.532, Natick, MA, USA) for preprocessing. Regions affected by solvent suppression were excluded (4.55–5.10 ppm for intracellular analysis and 4.58–4.90 ppm for extracellular analysis) and alignment procedures involving the correlation of optimized warping (COW) and interval correlation shifting (icoshift) algorithms were applied [41,42]. The spectra consisted of 8910 data points and were normalized using the probabilistic quotient method to overcome the issue of dilution [43].

The multivariate and statistical data analysis was performed on a set of the 32 assigned metabolites for intracellular metabolites and 27 metabolites for extracellular metabolites. All assignments were verified using the following databases: KEGG Pathways, PubChem, PAMDB, and ChenomX software (Chenomx Inc., Edmonton, AB, Canada). The concentration of metabolite measured by NMR was obtained as the sum of the intensities of the no overlapping resonances (or a part of partly overlapping resonances). The input for SIMCA-P software was a transformed data matrix (v 15.02, Umetrics, Umeå, Sweden). For additional analysis, Matlab was used. The data sets were unit variance scaled before the chemometric analysis. For bacteria strain classification, principal component analysis (PCA), and partial least square analysis (OPLS) were carried out. The multivariate data visualization marked an ellipse with Hotelling’s T2 range (95%). The OPLS-DA model reliability was tested with CV-ANOVA at the level of significance of α < 0.05. The most important variable was discrimination between comparisons, which was selected based on the variable importance in projection (VIP) value with a cutoff value of 1.00. Univariate analysis was performed using MATLAB software with Student’s *t*-test (equal/unequal variance) for data originating from a normal distribution and using Mann–Whitney–Wilcoxon test for data that did not meet this requirement. Normality of distribution was assessed by the Shapiro–Wilk test. The correction for multiple comparisons was preceded by the Benjamini–Hochberg procedure (FDR). All univariate statistics were carried out at the level of significance of α < 0.05.

### 5.6. Bioinformatics Analysis

After preprocessing, both, extra- and intracellular metabolite sets from two phenotypically different strains of *Pseudomonas aeruginosa* (antibiotic-resistant strain and antibiotic-susceptible strain) were used to perform metabolic pathway enrichment in the FELLA package [26]. FELLA is an R-package (public software) available in under the GPL-3 license [44]. To perform pathways, metabolites with statistical importance and VIP value > 1.00 were used. Firstly, KEGG-based hierarchical representations of biochemistry (knowledge graph) were built using *P. aeruginosa* PAO1 (T00035, Release 97.0+/03–04, Mar 21). Later, a list of metabolites from examined strains was separately mapped to the internal representation, creating an enriched object, and then subsequently the propagation algorithm was run using the diffusion method (undirected heat diffusion model) to score graph nodes. Additionally, the parametric z-score was computed using normality approximations for statistical normalization.

## Figures and Tables

**Table 1:** Antibiotic resistance of *Pseudomonas aeruginosa* strains (S—susceptible, R—resistant).

Antibiotic Name	Class	PAW17	PAW23
amikacin	aminoglycoside	S	R
gentamicin	aminoglycoside	S	R
netilmicin	aminoglycoside	S	R
tobramycin	aminoglycoside	S	R
imipenem	beta-lactam	S	R
meropenem	beta-lactam	S	R
piperacillin	beta-lactam	S	R
piperacillin/tazobactam	beta-lactam	S	R
ticarcillin/clavulanic acid	beta-lactam	S	R
ceftazidime	beta-lactam	S	R
cefepime	beta-lactam	S	R
ciprofloxacin	quinolone	S	S
levofloxacin	quinolone	S	R
colistin	polymyxin (peptide)	S	S

**Table 2:** The VIP score for discrimination OPLS with univariate analysis results for quantified metabolites in comparisons for intracellular metabolites *Pseudomonas aeruginosa* strains.

Compound Group	Metabolite	VIP Score for OPLS-DA Model	Mean/Median * Relative Concentration R	Mean/Median * Relative Concentration S	RSD R [%]	RSD S [%]	Fold Change R/S	*p* Value	FDR **
**Amino acids**	**Histidine**	1.238	0.030	0.022	7.096	12.502	1.376	6.22 × 10^−7^	6.63 × 10^−6^
	**Alanine**	1.282	0.206	0.152	11.021	8.278	1.358	3.23 × 10^−6^	1.72 × 10^−5^
	**Glutamate**	1.247	0.308	0.371	10.271	5.964	0.830	6.48 × 10^−5^	2.71 × 10^−4^
	**Valine**	1.246	0.185	0.140	13.759	9.833	1.323	1.06 × 10^−4^	3.40 × 10^−4^
	**Isoleucine**	1.322	0.095 ^#^	0.056 ^#^	18.228	11.873	1.692	1.83 × 10^−4^	4.87 × 10^−4^
	**Leucine**	1.359	0.383 ^#^	0.203 ^#^	18.323	8.907	1.887	1.83 × 10^−4^	4.87 × 10^−4^
	**Methionine**	0.912	0.022	0.015	25.325	20.409	1.453	3.18 × 10^−3^	7.27 × 10^−3^
	**Glycine**	1.029	0.202	0.229	10.051	7.502	0.881	4.38 × 10^−3^	8.76 × 10^−3^
	**Threonine**	1.034	0.042	0.038	10.142	5.496	1.109	1.62 × 10^−2^	2.88 × 10^−2^
	**Aspartate**	0.707	0.068	0.062	15.953	14.564	1.093	2.11 × 10^−1^	2.81 × 10^−1^
	**Phenylalanine**	0.705	0.113 ^#^	0.121 ^#^	14.269	7.127	0.933	5.21 × 10^−1^	6.02 × 10^−1^
	**Tyrosine**	0.508	0.115	0.118	10.702	10.218	0.970	5.26 × 10^−1^	6.02 × 10^−1^
**Amino acid** **metabolism**	**Homoserine**	1.358	0.619	0.392	9.718	5.487	1.578	1.85 × 10^−7^	2.96 × 10^−6^
	**Histamine**	1.300	0.016	0.009	16.267	16.722	1.725	1.50 × 10^−6^	1.13 × 10^−5^
	**Sarcosine**	1.048	0.022	0.016	19.383	28.508	1.386	5.94 × 10^−3^	1.12 × 10^−2^
	**5-aminopentanoate**	0.786	0.266	0.279	11.883	8.474	0.954	3.22 × 10^−1^	3.96 × 10^−1^
**Maim** **metabolism**	**Succinate**	1.382	0.669	0.401	9.236	7.523	1.669	1.43 × 10^−8^	4.57 × 10^−7^
	**Pyruvate**	1.231	0.199	0.238	10.392	5.611	0.834	7.63 × 10^−5^	2.71 × 10^−4^
	**Isocitrate**	0.885	0.169	0.193	16.536	3.586	0.873	2.18 × 10^−2^	3.51 × 10^−2^
	**Glucose**	0.602	0.031	0.036	25.176	14.910	0.864	1.22 × 10^−1^	1.77 × 10^−1^
**Cofactor**	**NAD+**	0.559	0.031	0.034	7.776	11.658	0.938	1.71 × 10^−1^	2.38 × 10^−1^
**Nucleotide processing pathways**	**UMP**	1.104	0.009	0.012	13.476	6.289	0.799	7.52 × 10^−5^	2.71 × 10^−4^
	**Adenine**	0.54	0.011 ^#^	0.010 ^#^	8.239	13.275	1.103	1.21 × 10^−1^	1.77 × 10^−1^
	**AMP**	0.797	0.067	0.072	12.569	12.192	0.932	2.21 × 10^−1^	2.83 × 10^−1^
	**Uracil**	0.364	0.010	0.010	26.399	19.769	0.964	7.31 × 10^−1^	8.06 × 10^−2^
**Others**	**Lactate**	1.284	0.274	0.186	12.175	11.863	1.471	1.76 × 10^−6^	1.13 × 10^−5^
	**Betaine**	1.083	8.428	7.200	11.685	7.209	1.171	2.63 × 10^−3^	6.47 × 10^−3^
	**Formate**	0.908	0.009	0.007	13.181	16.178	1.237	4.33 × 10^−3^	8.76 × 10^−3^
	**Ethanol**	0.991	0.075	0.065	14.898	6.110	1.153	2.19 × 10^−2^	3.51 × 10^−2^
	**Isobutyrate**	0.711	0.018 ^#^	0.017 ^#^	19.107	12.686	1.078	8.50 × 10^−1^	9.07 × 10^−1^
	**Oxypurinol**	0.1	0.034 ^#^	0.034 ^#^	9.793	9.363	0.997	9.10 × 10^−1^	9.39 × 10^−1^
	**Acetate**	0.59	0.066	0.065	13.588	25.511	1.006	9.48 × 10^−1^	9.48 × 10^−1^

R—drug-resistant *P. aeruginosa* strains. S—drug-susceptible *P. aeruginosa* strains; VIP (variable importance in projection) > 1.00 and statistically important metabolites are marked on the grey background; * mean—samples with normal distribution; median (marked with ^#^)—samples without normal distribution; ** FDR—Q values from false discovery rate control by Benjamini–Hochberg procedure.

**Table 3:** The VIP score for discrimination OPLS with univariate analysis results for quantified metabolites in comparisons for extracellular metabolites *Pseudomonas aeruginosa* strains (RC -relative concentration).

Group	Metabolites	Mean/Median RC R *	Mean/Median RC S *	Mean/Median RC C *	RSD R [%]	RSD R [%]	RSD K [%]	R vs. S				C vs. R				C vs. S			
								VIP Score	Fold Change R/S	*p* Value	FDR **	VIP Score	Fold Change C/R	*p* Value	FDR **	VIP Score	Fold Change C/S	*p* Value	FDR **
**Amino acids (AA)**	Threonine	6.583	4.868	6.128	3.623	5.620	1.900	1.197	1.352	1.37 × 10^−11^	8.24 × 10^−11^	0.886	0.931	1.57 × 10^−3^	2.22 × 10^−3^	1.083	1.259	2.49 × 10^−7^	7.46 × 10^−7^
	Leucine	8.607	6.050	10.65	2.352	8.221	5.513	1.201	1.423	4.14 × 10^−9^	1.99 × 10^−8^	1.133	1.237	1.03 × 10^−3^	1.54 × 10^−3^	1.118	1.76	6.48 × 10^−80^	2.22 × 10^−9^
	Isoleucine	2.676	2.191	2.516	4.134	8.724	8.159	1.115	1.221	1.75 × 10^−6^	6.00 × 10^−6^	0.584	0.94	6.76 × 10^−2^	8.54 × 10^−2^	0.881	1.148	9.65 × 10^−3^	1.22 × 10^−2^
	Glycine	0.093	0.138	1.155	16.681	11.456	1.660	1.080	0.678	5.93 × 10^−6^	1.78 × 10^−5^	1.203	12.363	5.73 × 10^−21^	4.93 × 10^−20^	1.14	8.387	1.10 × 10^−20^	1.78 × 10^−19^
	Phenylalanine	2.015	2.407	2.352	2.981	8.182	2.468	1.078	0.837	9.95 × 10^−5^	2.39 × 10^−4^	1.146	1.167	1.22 × 10^−7^	2.94 × 10^−7^	0.696	0.977	4.31 × 10^−1^	4.50 × 10^−1^
	Histidine	0.155	0.195	0.142	14.571	11.601	4.770	0.944	0.796	9.54 × 10^−4^	1.76 × 10^−3^	0.376	0.916	1.19 × 10^−1^	1.36 × 10^−1^	0.947	0.729	2.15 × 10^−5^	4.68 × 10^−5^
	Alanine	1.993	1.835	6.954	3.342	5.546	1.453	0.974	1.086	2.20 × 10^−3^	3.78 × 10^−3^	1.204	3.489	6.17 × 10^−21^	4.93 × 10^−20^	1.139	3.79	6.66 × 10^−4^	9.40 × 10^−4^
	Lysine	10.993	10.17	11.918	2.894	6.608	1.520	0.949	1.081	4.00 × 10^−3^	6.00 × 10^−3^	1.042	1.084	4.71 × 10^−5^	8.69 × 10^−5^	1.026	1.172	8.28 × 10^−6^	1.99 × 10^−5^
	Valine	3.985	4.177 ^#^	3.493	3.649	5.396	4.115	0.752	0.954	8.90 × 10^−2^	1.19 × 10^−1^	1.038	0.877	3.23 × 10^−5^	6.45 × 10^−5^	1.031	0.836	6.66 × 10^−4^	9.40 × 10^−4^
	Methionine	0.949	0.202 ^#^	1.205	5.045	97.014	1.313	0.943	4.706	1.40 × 10^−1^	1.61 × 10^−1^	1.154	1.27	2.82 × 10^−9^	9.66 × 10^−9^	0.943	5.977	1.27 × 10^−2^	1.52 × 10^−2^
	Tryptophan	0.078	0.026 ^#^	0.212	6.533	97.715	2.961	0.752	2.959	1.40 × 10^−1^	1.61 × 10^−1^	1.200	2.719	1.31 × 10^−15^	7.85 × 10^−15^	1.041	8.046	6.66 × 10^−4^	9.40 × 10^−4^
	Tyrosine	0.861	0.861 ^#^	0.599	4.827	15.001	0.640	0.817	1.000	4.73 × 10^−1^	4.73 × 10^−1^	1.160	0.696	6.50 × 10^−9^	1.73 × 10^−8^	1.022	0.696	6.66 × 10^−4^	9.40 × 10^−4^
**AA metabolism**	Histamine	0.283	0.167 ^#^	0.273	4.487	38.007	2.369	0.900	1.700	1.40 × 10^−1^	1.61 × 10^−1^	0.518	0.965	1.30 × 10^−1^	1.42 × 10^−1^	0.809	1.641	2.54 × 10^−1^	2.78 × 10^−1^
**Others**	Imidazole	0.078	0.047	0.055 ^#^	3.930	5.241	72.63	1.221	1.672	1.63 × 10^−15^	3.91 × 10^−14^	0.463	0.712	5.94 × 10^−1^	5.94 × 10^−1^	0.681	1.19	6.66 × 10^−4^	9.40 × 10^−4^
	Oxypurinol	0.096	0.100	0.226 ^#^	3.293	11.458	52.044	0.768	0.961	3.22 × 10^−1^	3.52 × 10^−1^	0.690	2.347	5.94 × 10^−1^	5.94 × 10^−1^	0.662	2.255	5.94 × 10^−1^	5.94 × 10^−1^
	Isobutyrate	0.436	0.692	0.204	6.416	3.999	19.06	1.221	0.630	5.94 × 10^−14^	7.12 × 10^−13^	1.164	0.468	5.73 × 10^−9^	1.72 × 10^−8^	1.134	0.295	4.70 × 10^−13^	2.82 × 10^−12^
	Acetate	3.486	10.284	2.589	32.191	7.080	2.005	1.200	0.339	4.04 × 10^−12^	3.23 × 10^−11^	0.522	0.743	3.24 × 10^−2^	4.32 × 10^−2^	1.135	0.252	6.94 × 10^−11^	2.77 × 10^−10^
	Pyruvate	0.184	0.153	0.256	5.221	5.938	1.400	1.131	1.203	7.16 × 10^−7^	2.86 × 10^−6^	1.178	1.388	7.05 × 10^−10^	2.82 × 10^−9^	1.129	1.669	3.91 × 10^−12^	1.88 × 10^−11^
	Betaine	5.167	5.667	6.108	4.576	4.925	1.544	0.970	0.912	4.17 × 10^−4^	9.10 × 10^−4^	1.111	1.182	1.26 × 10^−6^	2.74 × 10^−6^	0.818	1.078	4.91 × 10^−3^	6.55 × 10^−3^
	Formate	0.048	0.017	0.092 ^#^	40.445	24.975	49.725	0.989	2.816	6.25 × 10^−4^	1.25 × 10^−3^	0.58	1.924	7.53 × 10^−2^	9.03 × 10^−2^	0.94	5.419	7.53 × 10^−2^	8.60 × 10^−2^
	Trehalose	0.121 ^#^	0.084	0.317	27.213	7.319	3.308	0.744	1.444	3.85 × 10^−1^	4.01 × 10^−1^	1.174	2.613	6.66 × 10^−4^	1.07 × 10^−3^	1.137	3.773	8.92 × 10^−17^	7.14 × 10^−16^
	Methanol	0.035	0.043	0.062	6.492	13.301	2.112	0.859	0.826	2.45 × 10^−3^	3.92 × 10^−3^	1.193	1.741	5.12 × 10^−12^	2.46 × 10^−11^	1.03	1.439	9.90 × 10^−7^	2.64 × 10^−6^
	6-Hydroxynicotinate	0.113	0.126	0.054 ^#^	3.153	10.280	35.106	0.813	0.899	1.28 × 10^−2^	1.80 × 10^−2^	1.078	0.478	6.66 × 10^−4^	1.07 × 10^−8-3^	1.038	0.43	6.66 × 10^−4^	9.40 × 10^−4^

R—drug-resistant *P. aeruginosa* strains. S—drug-susceptible *P. aeruginosa* strains; VIP (variable importance in projection) > 1.00 and statistically important metabolites are marked on the grey background; * mean—samples with normal distribution; median (marked with ^#^)—samples without normal distribution; ** FDR—Q values from false discovery rate control by Benjamini–Hochberg procedure.

## Data Availability

MDPI Research Data Policies.

## Supplementary Materials

The following are available online at https://www.mdpi.com/article/10.3390/ijms221910820/s1. Table S1: ^1^H NMR signal assignments for intracellular metabolites. Table S2: ^1^H NMR signal assignments for extracellular metabolites. Figure S1: OPLS-DA score plot of ^1^H NMR data of *P. aeruginosa* strains. Drug-resistant extracellular (green), drug-susceptible extracellular (yellow), drug-resistant intracellular (blue), drug-susceptible intracellular (red). Figure S2: OPLS-DA score plot of ^1^H NMR data of intracellular metabolites of drug-resistant (blue squares) and drug-susceptible (red circle) *P. aeruginosa* strains. Figure S3: OPLS-DA models for ^1^H NMR data of extracellular metabolites of *P. aeruginosa* strains. (A) both *P. aeruginosa* strains with control; (B) both *P. aeruginosa* strains; (C) drug-resistant *P. aeruginosa* strain with control; (D) drug-susceptible *P. aeruginosa* strain with control. (drug-resistant extracellular (green squares), drug- susceptible extracellular (yellow circle), control—LB medium (gray triangles)). Figure S4: PCA model plots and corresponding loading plot for ^1^H NMR data of extracellular metabolites of *P. aeruginosa* strains. (A) drug-resistant *P. aeruginosa* strain with control (the first and second principal component (PC) accounted respectively for 67.6% and 11.8% of the total variance in the data (R2X = 0.864)); (B) drug-susceptible *P. aeruginosa* strain with control (the first and second principal component (PC) accounted respectively for 69.9% and 14.5% of the total variance in the data (R2X = 0.845)), (drug-resistant extracellular (green squares), drug- susceptible extracellular (yellow circle), control—LB medium (gray triangles)). File S1: Interactive version of results of the node prioritization by FELLA in the *Pseudomonas aeruginosa* strain (intracellular analysis). File S2: Interactive version of results of the node prioritization by FELLA in the *Pseudomonas aeruginosa* strain (extracellular analysis).
