# Supplementary material for: Metabolomics Comparison of Drug-Resistant and Drug-Susceptible Pseudomonas aeruginosa Strain (Intra- and Extracellular Analysis)
Source: Int J Mol Sci. 2021 Oct 6;22(19):10820. doi: 10.3390/ijms221910820 (PMC8509183; doi:10.3390/ijms221910820)
Supplement: Supplementary file 1 [file ijms-22-10820-s001.zip › Supplementary materials.pdf]

Supporting information

Table S1. ^{1}H NMR signal assignments for intracellular metabolites.

L.p.	Metabolites	KEGG number	Chemical shift [ppm]
1.	5-aminopentanoate	C00431	2.20 (t)
2.	Acetate	C00033	1.90 (s)
3.	Adenine	C00147	8.20 (d)
4.	Alanine	C00133	1.50 (d)
5.	AMP	C00360	8.60 (s)
6.	Aspartate	C00049	2.70 (dd)
7.	Betaine	C00719	3.30 (s)
8.	Ethanol	C00469	1.20 (t)
9.	Formate	C00058	8.40 (s)
10.	Glucose	C00031	5.20 (d)
11.	Glutamate	C00025	2.30 (m)
12.	Glycine	C00037	3.60 (s)
13.	Histamine	C00263	7.10 (s)
14.	Histidine	C00135	7.90 (s)
15.	Homoserine	C00065	2.00 (m)
16.	Isobutyrate	C06001	1.10 (d)
17.	Isocitrate	C00311	2.60 (m)
18.	Isoleucine	C00407	1.00 (d)
19.	Lactate	C00256	1.30 (d)
20.	Leucine	C00123	0.90 (t)
21.	Methionine	C00073	2.10 (s)
22.	NAD+	C00003	9.30 (s)
23.	Oxypurinol	C07599	8.20 (s)
24.	Phenylalanine	C00079	7.40 (t)
25.	Pyruvate	C00022	2.40 (s)
26.	Sarcosine	C00213	2.70 (s)
27.	Succinate	C00042	2.40 (s)
28.	Threonine	C00188	3.60 (d)
29.	Tyrosine	C00082	7.20 (d)
30.	UMP	C00105	8.10 (m)
31.	Uracil	C00106	5.80 (d)
32.	Valine	C00183	1.00 (d)

Table S2. ^{1}H NMR signal assignments for extracellular metabolites.

L.p.	Metabolites	KEGG number	Chemical shift [ppm]
1.	6-Hydroxynicotinate	C01020	8.10 (m)
2.	Acetate	C00033	1.90 (s)
3.	Alanine	C00133	1.50 (d)
4.	Betaine	C00719	3.30 (s)
5.	Formate	C00058	8.40 (s)
6.	Glycine	C00037	3.60 (s)
7.	Histamine	C00263	8.00 (s)
8.	Histidine	C00135	8.05 (s)
9.	Imidazole	C05568	8.20 (s)
10.	Isobutyrate	C06001	1.10 (d)
11.	Isoleucine	C00407	1.00 (d)
12.	Leucine	C00123	0.95 (t)
13.	Lysine	C00047	1.70 (m)
14.	Methanol	C00132	3.40 (s)
15.	Methionine	C00073	2.10 (s)
16.	Oxypurinol	C07599	8.25 (s)
17.	Phenylalanine	C00079	7.40 (m)
18.	Pyruvate	C00022	2.40 (s)
19.	Threonine	C00188	3.60 (d)
20.	Trehalose	C00689	5.20 (d)
21.	Tryptophan	C00078	7.70 (d)
22.	Tyrosine	C00082	6.90 (dd)
23.	Valine	C00183	1.05 (d)
24.	Glutamate	C00025	2.28 (m)
25.	Aspartate	C00049	2.82 (dd)
26.	Asparagine	C00152	2.99 (dd)
27.	Pyroglutamate	C01879	2.45 (m)

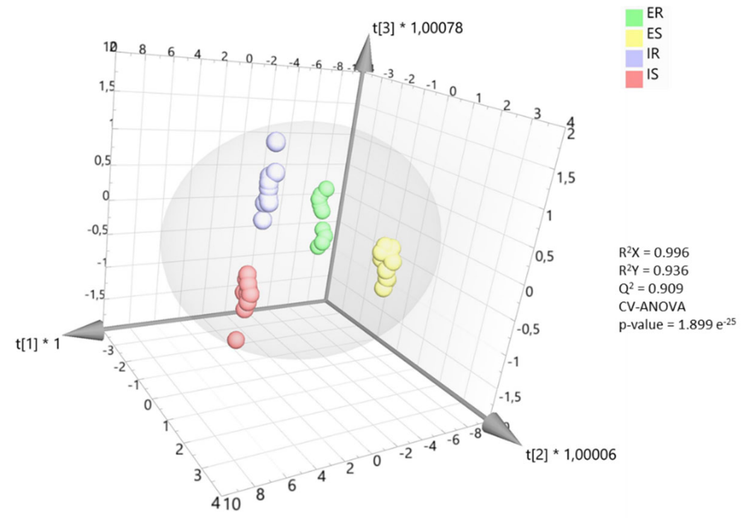

Figure S1. OPLS-DA score plot of ^{1}H NMR data of *P. aeruginosa* strains. (drug-resistance extracellular (green), drug-susceptible extracellular (yellow), drug-resistance intracellular (blue), drug-susceptible intracellular (red)).

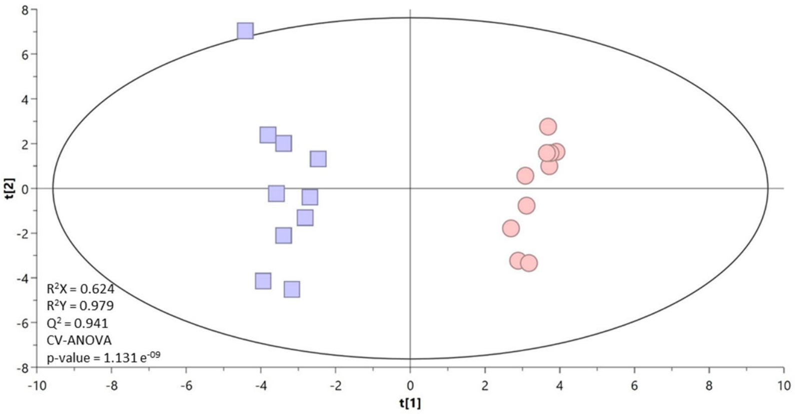

Figure S2. OPLS-DA score plot of ^{1}H NMR data of intracellular metabolites of drug-resistance (blue squares) and drug-susceptible (red circle) *P. aeruginosa* strains.

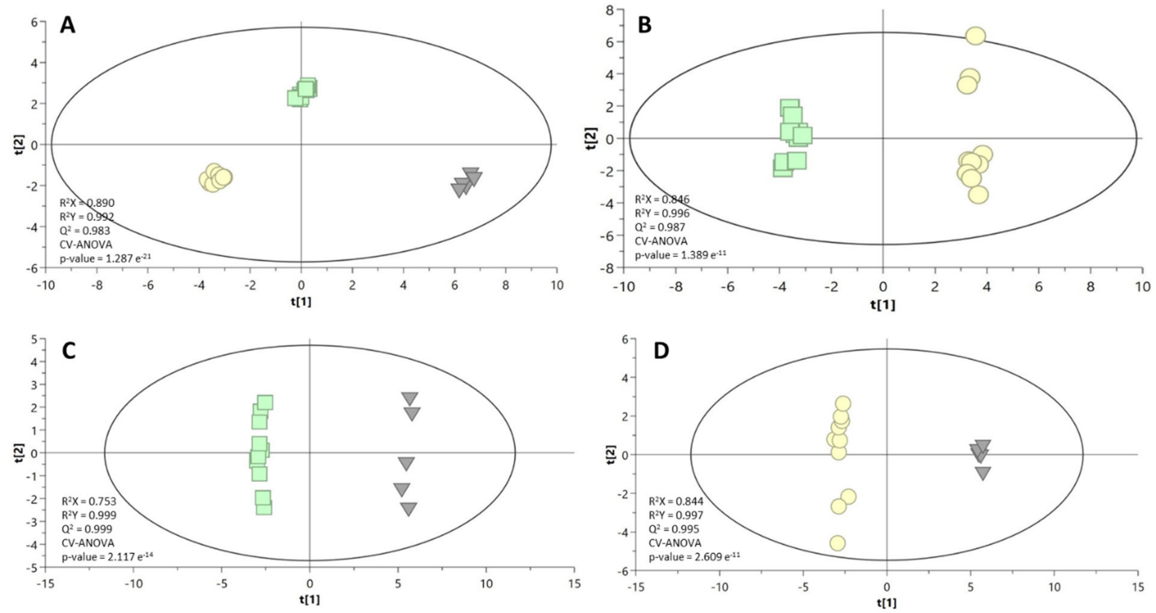

Figure S3. OPLS-DA models for ^{1}H NMR data of extracellular metabolites of *P. aeruginosa* strains. (A) both *P. aeruginosa* strains with control; (B) both *P. aeruginosa* strains; (C) drug-resistance *P. aeruginosa* strain with control; (D) drug-susceptible *P. aeruginosa* strain with control. (drug-resistance extracellular (green squares), drug- susceptible extracellular (yellow circle), control – LB medium (gray triangles))

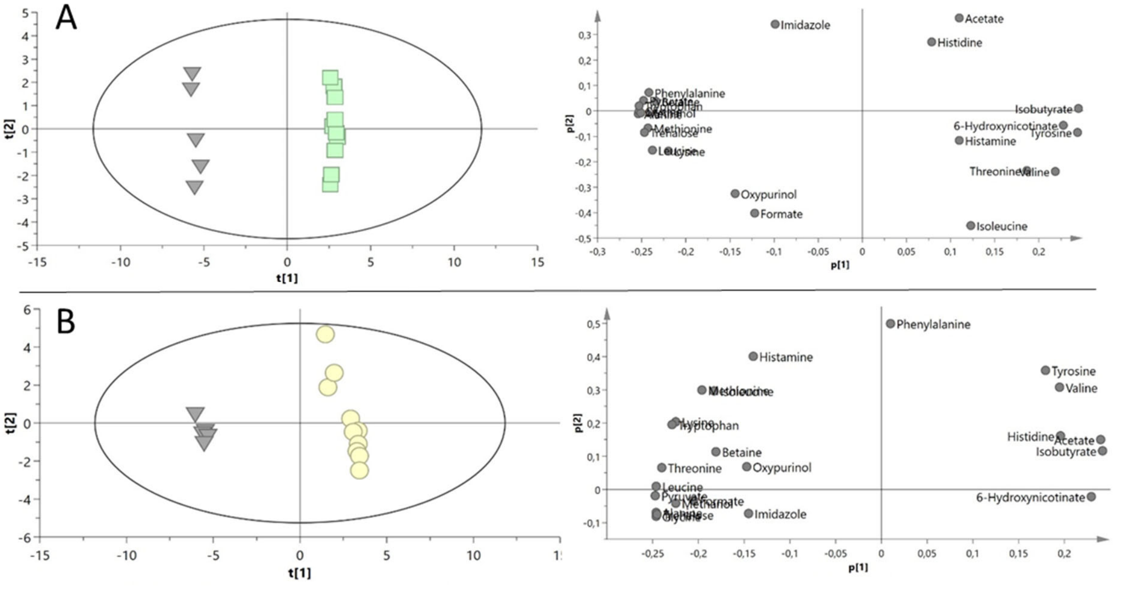

Fig S4. PCA model plots and corresponding loading plot for ^{1}H NMR data of extracellular metabolites of P. aeruginosa strains.(A) drug-resistance P. aeruginosa strain with control (the first and second principal component (PC) accounted respectively for 67.6 % and 11.8 % of the total variance in the data (R2X = 0.864, three PC's)); (B) drug-susceptible P. aeruginosa strain with control (the first and second principal component (PC) accounted respectively for 69.9 % and 14.5 % of the total variance in the data (R2X = 0.845, two PC's)). (drug-resistance extracellular (green squares), drug- susceptible extracellular (yellow circle), control – LB medium (gray triangles))
